# Bacterial Persister Cells and Development of Antibiotic Resistance in Chronic Infections: An Update

**DOI:** 10.3389/bjbs.2024.12958

**Published:** 2024-08-07

**Authors:** Anil Philip Kunnath, Mohamed Suodha Suoodh, Dinesh Kumar Chellappan, Jestin Chellian, Kishneth Palaniveloo

**Affiliations:** ^1^ Division of Applied Biomedical Science and Biotechnology, School of Health Sciences, International Medical University, Kuala Lumpur, Malaysia; ^2^ Department of Life Sciences, School of Pharmacy, International Medical University, Kuala Lumpur, Malaysia; ^3^ Institute of Ocean and Earth Sciences, Institute for Advanced Studies Building, Universiti Malaya, Kuala Lumpur, Malaysia

**Keywords:** bacterial persister cells, biofilms, chronic infections, antibiotic resistance, antibiotic tolerance

## Abstract

The global issue of antimicrobial resistance poses significant challenges to public health. The World Health Organization (WHO) has highlighted it as a major global health threat, causing an estimated 700,000 deaths worldwide. Understanding the multifaceted nature of antibiotic resistance is crucial for developing effective strategies. Several physiological and biochemical mechanisms are involved in the development of antibiotic resistance. Bacterial cells may escape the bactericidal actions of the drugs by entering a physiologically dormant state known as bacterial persistence. Recent findings in this field suggest that bacterial persistence can be one of the main sources of chronic infections. The antibiotic tolerance developed by the persister cells could tolerate high levels of antibiotics and may give rise to persister offspring. These persister offspring could be attributed to antibiotic resistance mechanisms, especially in chronic infections. This review attempts to shed light on persister-induced antibiotic resistance and the current therapeutic strategies.

## Introduction

Persister cells are a subset of bacteria that can survive high levels of antibiotics with genetic changes by reducing their metabolism and becoming metabolically inactive. When bacteria acquire genetic mutations or come into contact with an environment that restricts their growth, it leads to antibiotic tolerance [1]. The tolerance developed by persister cells is slow and reversible, allowing them to switch back to their active state once antibiotics are removed [2].

Several bacteria cause persistent infections, and the emergence of persister cells is a significant contributor to chronic infections and antibiotic resistance [1–3]. In recent times, it has been discovered that there are viable sub-populations of persister cells in fungal strains like Ca*ndida albicans* that can survive high concentrations of antifungal agents [4, 5]. This shows that persister cells have the ability to survive antibiotic treatments, which is a common feature among all microbial persisters. Essentially, bacterial persisters are phenotypic variants of regular cells that can survive harsh antimicrobial treatment. If the antimicrobial agent is removed, persister cells can resume their growth and could facilitate infection [3–5].

Formation of persister cells is not an inherited process and is mainly controlled by growth phases and various environmental stress factors. Persisters make up a small fraction of exponentially growing cells and do not reproduce in the presence of antibiotics. However, these cells switch back to an active state and generate the same variants as the original ones upon removal of the active drug [6]. They are usually obtained from cultures during the stationary phase, as nutrients may be limited in this period. By either acting as a viable reservoir for the emergence of resistant mutants or by promoting the acquisition of resistance elements, persisters accelerate the development of resistance [7].

The formation of persister bacterial cells is best described through DNA damage, which activates a stress response-dependent repair of the DNA. This process upregulates DNA repair functions and facilitates the persisters to survive in harsh conditions, including exposure to antibiotics. Environmental stressors activate this formation process, involving the main bacterial stress response pathways [8]. During subsequent infections, bacterial pathogens get exposed to different stressors in the host, causing them to develop into a “neither-grow nor die” state. This state helps them survive both antibiotics and the host’s immune responses, which can eventually lead to relapse in many infections. While antibiotic persisters have been extensively studied over the last decade, the focus has mainly been on how these bacteria survive exposure to antibiotics. The main areas that are not fully researched are the ability of drug-tolerant persisters to survive and their interaction with the host environment [9]. The formation of biofilms is a critical factor in bacterial persistence, making it difficult to eradicate infections. To combat persister cells, it is important to use agents that can eliminate biofilm matrix or hinder biofilm development [8]. Study findings in *Streptococcus* suis, a zoonotic agent that causes sepsis and meningitis in pigs and humans, show that target-modifying enzymes and mutations in antibiotic targets lead to resistance [6]. Sequencing analysis on *Escherichia coli* revealed that carbon and energy metabolism genes were involved in the development of antibiotic resistance [7].

A wide range of bacteria is responsible for persistent infections, including *Mycobacterium tuberculosis*, *Salmonella enterica*, *Chlamydia pneumoniae*, *Brucella abortus*, *Borrelia burgdorferi*, *Pseudomonas aeruginosa*, pathogenic *E. coli*, *Klebsiella pneumoniae*, and *Streptococcus pneumoniae* [10, 11]. However, for long, there was little understanding of the significant relationship between the persisters and persistent infections until several researchers discovered high persister mutants in patients with cystic fibrosis (CF) who had undergone repeated courses of antibiotic therapies. For example, studies showed that, in cystic fibrosis bacterial mutations in the mucA gene lead to the mucoid phenotype, characterized by overproduction of alginate which protects the bacteria from antibiotics and immune responses and mutations in the mexT gene. These events lead to the upregulation of the MexEF-OprN efflux pump, contributing to antibiotic resistance. LasR mutations affect quorum sensing and virulence, aiding in evasion of the immune system. rpoN mutations affect nitrogen metabolism and contribute to persistence in the nutrient-limited environment of the CF lung. These findings have shed light on the possibility of bacterial persisters and their offspring developing antibiotic resistance [12–17].

## Antibiotic Tolerance of BIOFILMS and Persister Cell Formation

One of the usual survival strategies employed by bacteria is to adhere to each other, forming a dynamic structure called biofilm, within a self-produced matrix that is resistant to antibiotics and the host’s immune clearance. A biofilm may protect the bacterial cells from the host immune system, serving as a protective barrier that confers tolerance against complement immunity and phagocytosis [18, 19]. The antibiotic tolerance of a microbial biofilm is thought to be central to the treatment failures in biofilm associated diseases [20]. Bacteria can alter conditions using quorum-sensing (QS) systems by increasing cell density, playing a crucial role in biofilm development. Recent studies have demonstrated that mutations in cell density-sensing (quorum-sensing) systems have become a significant factor contributing to biofilm-associated infections in *Staphylococcus aureus*, a prominent human pathogen [21].

Extracellular polymeric substances (EPS) have been widely reported to be the primary factor for the formation of physical and social interactions in biofilms, facilitating horizontal gene transfer, and antimicrobial tolerance. EPS largely consists of cellulose, alginates, poly-N-acetyl glucosamine, extracellular teichoic acid, proteins, lipids, nucleic acids, phospholipids, polysaccharides, extracellular DNA, and other organic compounds. About 90% of the biofilm’s biomass is composed of EPS, which typically possesses a mushroom-like structure [22–24]. EPS plays a major role in bacterial protection, structure, recognition, adhesion, and physiology. These factors highly influence the transformative capability of antibiotic resistance genes [23].

It has been observed that bacterial persistence and biofilm formation are both key factors in multidrug tolerance. While resistance mechanisms aim to prevent antibiotics from reaching their targets, tolerance works by shutting down those targets. While bactericidal antibiotics work by corrupting the targets, shutting them down may actually protect bacteria from being killed. As bacteria move from exponential growth to slow or no growth, they become more tolerant to antibiotics. Typically, the number of persisters in a growing population of bacteria increases during mid-log and reaches its peak during the stationary phase. It has also been observed that mature biofilms have slow-growing bacteria. The proportion of persister cells is low during the log phase which increases significantly in the stationary, and death phases, making eradication a challenge [25].

Understanding these mechanisms can help in developing better strategies for combating drug-resistant bacteria [26–30]. Bacterial biofilms can be seen as a specific form of persistent bacterial infection, given that they contain persister cells that are highly concentrated in the biofilm. It is estimated that over 65% of all infections are associated with biofilms, which are difficult to eliminate through conventional antimicrobial methods due to their elevated resistance [31]. Microbial populations benefit from the physical barrier that biofilms provide. However, when nutrients are limited, metabolic dormancy is an option for the entire population and can lead to tolerant persister cells. This subpopulation of cells in a state of dormancy confers benefits to the general cell population and is responsible for the high resistance of bacterial biofilms to antimicrobial agents. Genetic diseases such as cystic fibrosis are vulnerable to respiratory infections that can involve the development of biofilms in the lungs, infective endocarditis, wound infections, and infections of indwelling devices, are highly challenging to treat with antibiotics because of biofilm production and may result in chronic bacterial infections that are difficult to eradicate, or in some cases, require the removal and replacement of an infected device or debridement of an infected wound [31–35]. Recent research has indicated the potential emergence of antibiotic-resistant mutants within biofilm populations of *E. coli* strain LF82 subsequent to treatment with amikacin, a recommended treatment for *E. coli* infections. The study showcased that intermittent antibiotic treatment is associated with heightened mutation rates in sbmA, responsible for encoding an inner membrane peptide transporter, and fusA, responsible for encoding the essential elongation factor G. These findings shed light on the importance of exploring alternative treatment strategies to combat biofilm-associated tolerance [36]. Furthermore, biofilms offer protection for internal bacteria against harsh environments such as metal toxicity, acid exposure, dehydration, and salinization, in addition to the barrier effect [37–40].

In another study, *E. coli* biofilms were used to identify genes involved in biofilm tolerance to fluoroquinolone and ofloxacin. Generally, amino acid auxotrophs exhibit higher levels of tolerance that are specific to biofilms, and antibiotic tolerance phenotype is largely influenced by starvation. The study also discovered that the SOS response played a significant role in ofloxacin tolerance in biofilm-associated persisters [41, 42]. For example, *P. aeruginosa* is a pathogen that exploits immunocompromised patients. It can lead to incurable infections associated with biofilms in cystic fibrosis (CF) patients. High-persister (hip) mutants appeared in 10 of the 14 instances that were studied in multiple CF patients. As disease progressed, repeated exposure of the bacterial population to antibiotics resulted in further formation of persister cells. This clearly demonstrates the direct correlation between the persisters in *P. aeruginosa* biofilm infections in the cystic fibrosis lung and the resistance of this infection to antimicrobial treatment [43].

An investigation was conducted on cancer patients who were infected with oral *Candida albicans*, the fungus responsible for causing oral thrush. The fungus creates oral biofilms that can be challenging to treat. The study examined longitudinal isolates of patients who were treated with chlorhexidine daily and were divided into two groups: transient carriers and long-term carriers. The study found that only long-term carriers had Hip mutants, which confirms the link between persisters and drug tolerance [44]. Additionally, a recent study on a new anti-persister antibiotic treatment further confirms the role of persister cells in biofilm tolerance. The study showed that the dispersion of nutrients and oxygen within the biofilm hinders antibiotic penetration and favors the development of antibiotic tolerance and bacterial persistence [45]. The ability to target and kill persisters directly translates to the ability to kill the biofilm [5, 19]. A separate study further revealed a correlation between antibiotic persistence and cell wall deficient bacteria (CWDB), which includes spheroplasts and L-form bacteria produced by beta lactam antibiotics [40].

## Antibiotic Resistant Persister Offspring Generation From Persister Cells

Biofilms are communities of microorganisms that exhibit unique biological properties when compared to their free-floating counterparts known as planktonic organisms. Within bacterial communities, intricate genetic and phenotypic mechanisms give rise to persistent progeny that are resistant to antibiotics. Researchers have observed that the development of biofilms is directly linked to persistent infections, particularly in cases of chronic colonization of *P. aeruginosa* in the lungs of cystic fibrosis patients. Once a biofilm is formed, the bacteria within the community develop the ability to survive various types of physicochemical assaults, such as UV light, heavy metals, acidity, changes in hydration or salinity, and phagocytosis. In addition, biofilm bacteria display an inherent ability to resist the effects of antibiotics, which poses significant therapeutic challenges in clinical settings. This phenomenon is referred to as the “recalcitrance” of biofilm bacteria towards antibiotics, which results from a complex interplay of several tolerance and resistance mechanisms.

Resistance to antibiotics can be either genetically inherited or acquired through horizontal gene transfer. For example, mutations in genes encoding antibiotic targets such as ribosomal proteins, DNA gyrase can reduce antibiotic binding. Overall, the unique properties of biofilms make them a significant source of therapeutic challenges and a persistent problem in clinical settings [40, 45–48]. Recent studies conducted on the ability of bacterial biofilms to survive high concentrations of antibiotics led to a complete shift in our understanding about the mechanisms involved in biofilm recalcitrance. In the past, it was thought that the biofilm’s resistance was due to the extracellular matrix (ECM) that surrounded the bacteria. Various research studies have suggested that the biofilm matrix’s mechanical and physicochemical characteristics might hinder the penetration of several substances, such as antibiotics and antiseptics, or delay their effect. For instance, the effect of an antibiotic may be reduced after its adsorption on the matrix due to electrical interactions with polymers surrounding the biofilm bacteria [49–53].

Antibiotics such as aminoglycosides which are positively charged and highly effective against a wide range of bacterial infections but several studies have suggested that the passage of aminoglycosides through negatively charged polymers of the biofilm matrix is relatively slow. This is because the extra polymeric substance matrix of biofilm contains negatively charged components such as extracellular DNA and certain polysaccharides. These negatively charged components can bind to the positively charged aminoglycosides, which reduces their free concentration and slowing their passage through the biofilm. In this regard, the chemical structure of the biofilm matrix becomes significant, and it has been shown that, even for a single pathogen, different types of exopolysaccharides may be involved, depending on the environment surrounding the biofilm. In contrast, the phenotypic outcomes of antibiotic penetration delay could be significant. For example, a bacterial cell may adapt to antibiotics through metabolic or transcriptional alterations induced by antibiotic stress. Additionally, due to their slow diffusion rate, biofilm bacteria may be temporarily subjected to sub-inhibitory concentrations of antibiotics. The subinhibitory levels of antibiotics may promote genetic variability by increasing the rate of mutation, recombination and horizontal gene transfer, quorum sensing which together accelerate the emergence and spread of antibiotic-resistant persister offspring from the biofilms [54–59]. Studies showed the maintenance of biofilms using quorum sensing signals helps maintain their structural integrity and resilience. The current research is aimed at developing quorum sensing inhibitors to combat antibiotic resistance [58].

Furthermore, the molecular mechanisms involved in the persister cell formation have been most extensively studied and characterized in planktonic cells. It is important not to assume that mechanisms of persister formation in planktonic and biofilm cultures are identical, since planktonic and biofilm cultures represent distinct physiological forms. Most of the laboratory analyses on biofilm-specific antibiotic resistance and tolerance mechanisms have mainly focused on single-species biofilms. However, it is becoming increasingly clear that interactions between different species in a biofilm can modulate the overall antimicrobial tolerance of the community, and that these multispecies interactions may be clinically relevant as many infections are polymicrobial in origin [60].

## Toxin-Antitoxin System and Its Significance in Phenotypic Switching in Biofilms

Bacterial toxin-antitoxin systems (TAs) are a set of genetic elements present in bacterial cells that encode for a toxin and its corresponding antitoxin. These systems were initially associated with plasmid maintenance, where they ensure the stable inheritance of plasmids during cell division. However, researchers have discovered that these TAs play a crucial role in the development of bacterial resistance and the formation of persistence [57].

Bacterial plasmids contain genes for toxins and antitoxins, which play a crucial role in maintaining stability within the host cell. Toxins and antitoxins work together and form complexes to deactivate the toxins. The faster breakdown of antitoxins compared to toxins necessitates the continuous expression of toxin-antitoxin genes to control the toxins effectively. When plasmids are lost during cell division, the expression of toxin-antitoxin genes is disrupted. In the absence of these genes, the decrease in antitoxin levels leads to the release of free toxins, inhibiting cell growth. This dynamic can promote diverse growth behaviors in bacterial subpopulations, contributing to a more varied and adaptable bacterial population [61].

In terms of resistance, TAs provide a mechanism for bacteria to survive in hostile environments such as those created by antibiotics. When exposed to antibiotics, bacterial cells with certain TAs have the ability to enter a dormant or persister state, allowing them to survive until the antibiotic is removed. This is accomplished by the toxin component of the TA system, which interferes with vital cellular processes, and the antitoxin component, which neutralizes the toxin. In the presence of antibiotics, the antitoxin is degraded, allowing the toxin to be released and trigger the persister state [61]. In addition the recent studies showed that free toxin triggers molecular stripping of the repressor complex off the DNA through multiple allosteric changes causing DNA distortion and ultimately leading to derepression [62].

In addition TAs have been shown to be involved in the formation of persisters by inducing a state of reversible growth arrest in bacterial cells. This allows the cells to conserve energy and withstand a wide range of environmental stresses [63, 64].

The TA system comprises two genetic elements that are closely linked, and most of these genes exist together as operons, resulting in transcriptional coupling. Recently, TA modules have been categorized into eight types based on how the anti-toxin regulates the toxin. Type-I TA system is a protein toxin that inhibits translation by cleaving mRNA and an antisense RNA is the antitoxin that inhibits the translation from mRNA of the toxin. In type II, both antitoxin and toxin are proteinaceous, allowing antitoxin proteins to directly bind and inhibit the toxin protein. In type III, RNA antitoxins bind with toxin proteins and neutralize them by forming pseudo-knots. Type IV antitoxin proteins stabilize the bacterial filaments, while the toxin causes destabilization of these elements. In type-V, protein RNAse antitoxins cleave the mRNA that encodes the toxin protein. In type-VI TA systems, the antitoxin protein, with protease adapters, degrades the toxin protein by providing it with a cellular protease. Type VII toxin protein that interferes with cell wall synthesis and antitoxin protein that neutralizes the toxin. Type VIII protein toxin with nuclease activity that targets RNA and antitoxin protein that binds and neutralizes the toxin [62, 65–69].

The TA system induces dormancy in bacteria due to external stimuli and events that degrade antitoxins. These include antibiotics, starvation, pH/temperature extremes, and host immune systems. These stresses activate toxins by over-expression or antitoxin degradation [70, 71].

In 1983, the identification of hip mutants provided the first ever evidence of a direct relationship between the TA system and persister formation. The interaction between the two substitutes of the HipA7 toxin and the anti-toxin may be poor due to the structures of HipAB, which enhances the persister formation activity. In a study conducted *in vitro* experiments that revealed overexpression of several toxins increases bacterial persistence [72]. Another study reported that the deletion of ten endoribonuclease coding TA systems, such as RelE, YoeB, HigB, YhaV, YafO, YafQ, MazF, ChpB, MqsR, and HicA from the *E. coli* chromosome, resulted in reduced persistence [73]. The results suggest that the TA loci are responsible for 99% of persisters formed during the exponential phase, while less than 1% arise from other random growth phases. Controlling transcription and expression of the TA gene is one of the ways to prevent toxin activation, which can also regulate the entry of bacterial cells into dormancy [72–76]. The recent studies shows that interactions between hosts and bacterial mobile genetic elements plays a crucial role in the conformational behaviour and stability of antitoxins [74].

The MqsRA gene in *E. coli* is the first TA (toxin-antitoxin) system that has been linked with biofilm formation. Additionally, this gene has been found to have a direct correlation with persister cell formation. When the mqsR locus is deleted from the mqsRA system, it reduces persister formation. On the other hand, when MqsRA is produced, it increases persistence [77–79]. This gene inhibits translation and prevents the stress response mechanism. It also relies on CspD and Hha to form persister cells. In comparison to non-persisters, this gene is the most effective in persister cells. Other toxins, such as MazE, ChpS, and YefM, behave similarly. Furthermore, the deletion of the tisB operon can have a significant effect on the level of persistence. Overexpression of the TisB toxin in the exponential phase can induce persistence, indicating that bacteria can enter a persistent state in several ways. Recently, Brown, in a report, revealed that nitrogen starvation in *E. coli* can also induce persister formation. NtrC modulates the transcription of the relA gene in N-starved *E. coli* [80–82]. Likewise, inactivation of relE toxin genes can affect the dormancy in *M. tuberculosis*. Moreover, VapC type II toxin overproduction causes dormant cell induction in *Mycobacterium smegmatis*. Even in *salmonella* infections, TA systems have exhibited persistence induction [83–88].

An increase in the level of (p)ppGpp alarm is also linked to the formation of persisters. The production of (p)ppGpp is typically a stress response that inhibits translation. An increase in the level of (p)ppGpp can also increase the level of inorganic polyphosphate [poly(P)], which can activate the proteases known as Lon. Lon degrades the antitoxins, which increases the toxicity, leading to the formation of a metabolically inactive cell. Recent studies have developed a mechanism that discusses the relationship between TA and (p)ppGpp. It has been found that persisters can still be formed without the presence of Lon or (p)ppGpp [89–92]. Based on this evidence, it has been hypothesized that TAs play a central role that is advantageous for cell survival in their natural habitat. The toxins in TAs inhibit cell growth by targeting important cellular processes, such as DNA replication, transcription, and cell wall synthesis. These processes are similar to antibiotic activities [93–96].

The bistable nature of toxin/antitoxin (TA) modules can cause the phenotypic switch between normal and persister cells. Under the same conditions, a bistable system can exhibit one of two stable behaviors, leading to heterogeneous populations of cells depending on the cell environments. The frequency of persisters is tuned by the overall number of toxin-antitoxin systems in the cell, using the growth rate as the coordinating signal. Toxin and antitoxin genes are closely linked and are capable of transferring from one genome to another through horizontal gene transfer [96–99].

## Elimination Strategies Against Persister Cells and Treating Biofilm Infection

The biofilm’s antimicrobial tolerance is attributed to the presence of dormant persister cells that evade conventional antibiotics. The resilience and adaptability of bacteria in various situations needs a multimodal approach to eradicate persister cells and biofilm infections. The conventional antibiotics rely on corrupting active processes in the cell. One strategy for treating biofilm-based infections is enzymatic degradation of the biofilm matrix. Enzymes that disrupt biofilms, including dispersin B, proteases, or DNase, can break down the biofilm matrix, increase the penetration of antibiotics and decrease the environment that shields persister cells [100–102].

Allison *et al.*, have proposed a mechanism to eliminate persisters within a biofilm through metabolite stimuli [103]. Persister cells exhibit aminoglycoside tolerance, primarily due to their low membrane potential.

A proton motive force is required for the uptake of aminoglycosides, which is lacking in persister cells. However, the introduction of specific sugars into the culture induces the uptake of gentamicin and generates a proton motive force, leading to persister cell death. This method has been shown to be effective not only against *E. coli* but also against *S. aureus*. The addition of mannitol (for *E. coli*) or fructose (for *S. aureus*) in the treatment regimen has enabled the eradication of biofilms in a mouse catheter infection model. More recent studies have demonstrated that L-arginine also facilitates biofilm eradication by gentamicin by affecting the pH. The efficacy of treatment has been demonstrated in a mouse catheter model of infection [103, 104].

The use of phage-antibiotic combinations to treat bacterial infections is becoming more popular because of the frequently noted synergistic benefits of using both to eliminate the persistor cells. According one study phage-antibiotic combinations may lessen the likelihood that bacteria would develop an antibiotic resistance [105]. Another approach is to find antimicrobial nanomaterials that are useful in improving biofilm penetration and subsequently eradicating persistor cells within the biofilm. New technologies, like the CRISPR-Cas system, can also be used to precisely eradicate certain bacterial populations by focusing on and rupturing vital genes in persister cells [106].

For the treatment of biofilm driven infections, antimicrobial peptides (AMPs) show promises as a novel anti-infective drug. In combination with tobramycin, a study that examined the effectiveness of AMP temporin G (TG) against preformed *S. aureus* biofilm, including persisters, revealed a 50%–100% reduction in biofilm viability [107].

A persister-specific compound called 3-[4-(4-methoxyphenyl)piperazin-1-yl]piperidin-4-yl biphenyl-4-carboxylate (C10), which was identified while screening 6,800 chemicals in a random chemical library, which showed dramatically reduced persistence frequency with fluoroquinolone antibiotics for both *E. coli* and *P. aeruginosa*, as reported by Kim *et al.* Persister cells rely on stress response pathways to survive hostile conditions, including antibiotic treatment. C10 inhibits key stress response regulators, such as toxin-antitoxin systems or global regulators like ppGpp (guanosine tetraphosphate), which are crucial for persister cell survival [39].

To improve the effectiveness of antibiotics in eradicating biofilms, they can be used in conjunction with adjuvants that boost antibiotic efficiency. Efflux pump inhibitors, for instance, have the ability to raise antibiotic intracellular concentrations. In a validated mouse model, a study employing gold nanocluster adjuvant shown improved lethality of up to 4 orders of magnitude [108, 109].

ADEP4 is an antibiotic that is synthetic and belongs to the acyldepsipeptides class. It works by binding the ClpP protease, which opens up its proteolytic core, leading to dysregulation of proteolysis. In another experiment, the combination of ADEP4 with rifampicin completely eradicated the population up to the limit of detection in various experiments, including a *S. aureus* biofilm [110–113].

To eliminate dormant persister cells in biofilms, it is necessary to employ specific approaches that involve the use of compounds which can penetrate the cell membrane without requiring any active transport. This approach has been proposed as a promising strategy for targeting persister cells within biofilms [114].

## Chronic Infections and Antibiotic Resistance Are Potential Outcomes of Bacterial Persistence

The fact that persister cells are resistant to both host immune responses and antibiotic therapy contributes significantly to their importance in the treatment of chronic infections [115]. Biofilms, which comprise several ecological niches and phenotypes, are home to over 99% of bacteria in natural populations. As a result, we are unable to completely eradicate persistent infections. Regrettably, the development of antibiotics has focused only on the planktonic minority, which is linked to systemic disease.

The diverse microbial species come together to form the biofilm, attaching to tissues or organs and diffusing and injecting agents through multiple secretion systems. Depending on the anatomical site of the infection, the persistor bacteria employ different techniques to subdue the host defense systems. It can form on medical devices such as catheters and orthopedic prostheses, as well as within tissues.

Unfortunately, treating bacterial infections can be a lengthy process for certain diseases. A prime example of this is tuberculosis, where the standard treatment lasts between six and 24 months. This extended treatment duration is necessary because of the risk of relapse. If bacterial populations are reduced to below the detection limit but are not eliminated, bacteria may regrow, leading to treatment failure. This is a significant barrier in reducing treatment length and requires careful monitoring and management of the patient’s condition [116, 117].

When an infection affects the body, the host’s innate immune system usually serves as the first line of defense. As a result, the host develops defenses to identify the presence of bacteria in tissue using an innate immune surveillance system that can identify molecular patterns linked to pathogens. These pathogen recognition receptors, known as Toll-like receptors (TLRs) or nucleotide oligomerization domain (NOD)-like receptors, are found in the cytosol and can detect products that are thought to be specific to bacteria, such as flagellin, lipopolysaccharides (LPS), lipoproteins, lipoteichoic acids, and lipoproteins. This detection can trigger the initial proinflammatory response. Nonetheless, persistent infections have developed both passive and active defense strategies to avoid being recognized by TLRs and innate immune system NOD-like receptors [118].

**FIGURE 1:**
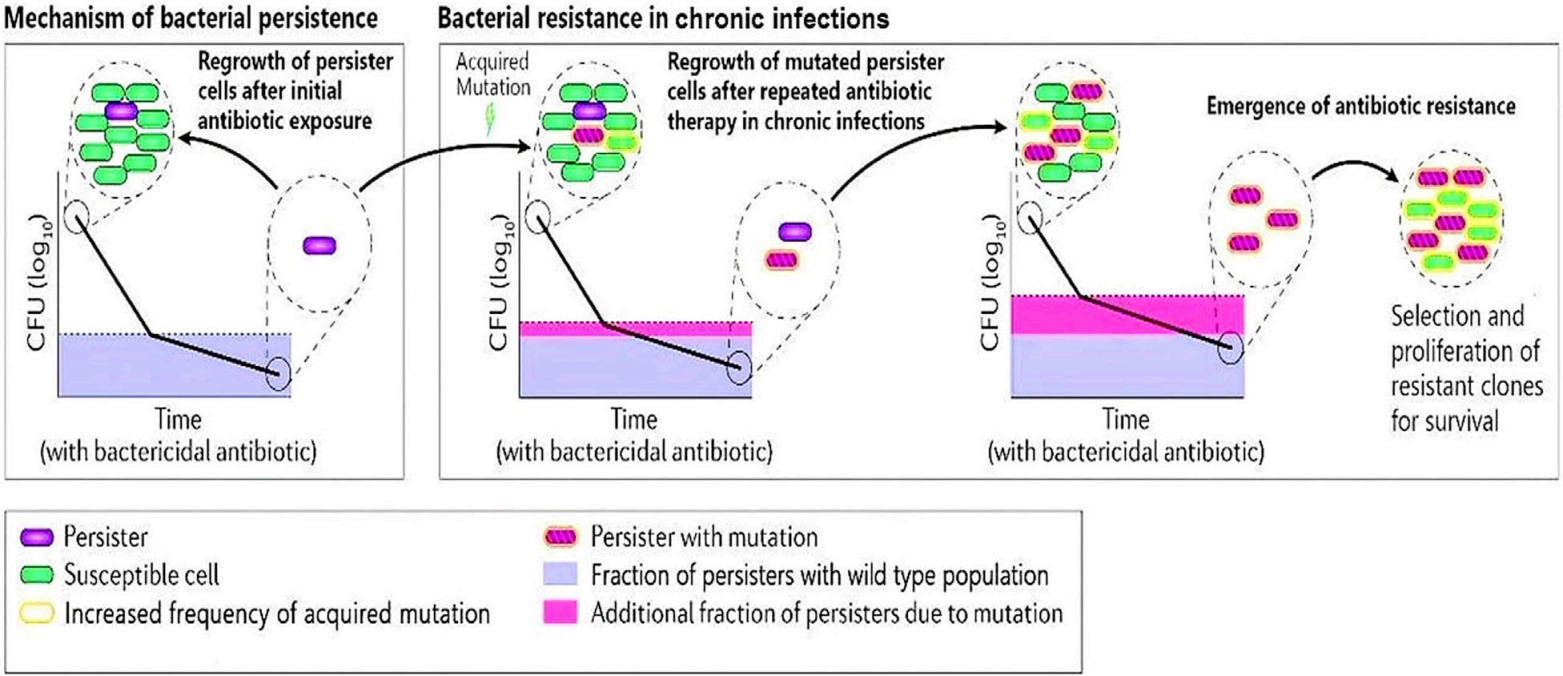
A schematic diagram describing bacterial persister cells undergoing acquired mutations after repeated antibiotic therapy in chronic infections. The clone of resistant cells will undergo selective proliferation and develop antibiotic resistance.

Numerous studies have shown that the persistence of bacterial cells, which is characterized by antibiotic tolerance, not only leads to treatment failure but also promotes the evolution of resistance (Figure 1).

A recent study among post-menopausal population indicated bacterial persistence in uropathogens from urogynecology patients despite treatment with commonly prescribed antibiotics [119]. It is also important to remember that biofilms and persister cells can cause chronic inflammation. Tissue damage and inflammation will arise from the immune system’s ongoing response to this ongoing infection.

## Future Treatment Strategies

The phenomenon of bacterial persistence is believed to be a significant factor in the failure of antibiotic therapy in clinical settings. Bacteria can use a broad variety of fundamentally distinct resistance mechanisms, which determine their ability to survive antibiotic therapy [120].

Studies have indicated that tolerant bacteria and persister cells can act as bacterial reservoirs, leading to the emergence of antibiotic resistance. Thus, it is crucial to develop a better understanding of the formation of persister cells and devise new strategies to address this issue effectively. One approach to eradicating persisters involves gaining insight into how growth-arrested persisters regrow and increase drug susceptibility through combination therapy. Furthermore, persister cells exhibit differences in their patterns of regrowth and varying levels of heterogeneity within the sub-population, making it challenging to develop a comprehensive understanding of this phenomenon. Additionally, it remains unclear how the cellular pool of antitoxin is replenished when the toxin inhibits translation [121–123].

Recent studies have shown that bacterial persistence is a complex phenomenon that is influenced by various external factors, such as physical stresses and nutrient availability. Over the past decade, numerous studies have identified multiple processes that contribute to bacterial persistence. Given the physiological complexity of each bacterial cell, it is possible that persistence may arise from fluctuations in various tolerance-associated processes. As a result, it is believed that within a single bacterial population, there may be many different types of persisters, each with distinct mechanisms for evading the lethal effects of bactericidal antibiotics. To test this hypothesis and further understand persister physiology, advances in sequencing and single-cell technologies are being developed. These technologies will help researchers to better elucidate the complex mechanisms underlying bacterial persistence and aid in the development of standard techniques for studying persister physiology. With the help of these advancements, we can gain a more comprehensive understanding of bacterial persistence and develop new strategies for combating antibiotic resistance [124–126].

It is now known that the regrowth of persisters, which are strongly linked to stress responses, may lead to mutations and adaptations in bacterial populations. It is possible that the offspring of persisters, which are often expressed at higher levels of DNA repair proteins like error-prone polymerases, can increase the rate of mutation during replication. This, in turn, theoretically increases the likelihood of antibiotic resistance emergence in persistent infections, especially if the drug is still present in low levels in the host [10, 127–129]. Additionally, the SOS response, which is known to induce the formation of persisters, can also upregulate the mechanisms of horizontal gene transfer, such as conjugation and phage activation. This can further promote the spread of antibiotic resistance following persister regrowth. Furthermore, combining evolution experiments with close monitoring of antibiotic tolerance and resistance in populations of *E. coli* has revealed that mutations leading to resistance to antibiotics like ampicillin occur after mutations that increase antibiotic tolerance. These findings suggest that tolerant bacteria and persister cells serve as important reservoirs for the emergence of antibiotic resistance [130–132].

Bactericidal antibiotics kill bacteria by affecting certain cellular targets that are necessary for active growth. For instance, ampicillin inhibits cell-wall synthesizing enzymes, which leads to bacterial death. However, there is another approach to combat bacterial infections, which involves using antibacterial compounds that target cellular components not only in actively growing cells but also in resting cells. Acyldeptipeptides are a class of antibiotics that activate ClpP, a core unit of a bacterial protease complex present in many bacteria. These antibiotics bind directly to ClpP and unleash unregulated and lethal protein degradation. It is believed that this proteolytic cascade should be lethal for both growing and non-growing bacteria. Researchers tested this hypothesis and found that one such acyldeptipeptide (ADEP4) was able to completely eradicate *S. aureus* biofilms *in vitro*. Moreover, when administered together with a conventional antibiotic, ADEP4 cleared a severe biofilm infection caused by *S. aureus* in mice [97, 105, 133].

In order to combat the problem of persister cells, which are dormant and resistant to traditional antibiotics, new therapeutic approaches are required. One possible approach is to develop methods to enter the persister cell without the need for active transport [97]. Researchers are currently searching for potential inhibitors to inihibit ppGpp synthesis and Inhibitors of bacterial efflux pumps to combat this problem. Synthetic ppGpp analogs have been developed that effectively inhibit the ppGpp synthetase activity of Rel proteins, which interferes with the long-term survival of Gram-positive bacteria [134].

## Conclusion

Antibiotic resistance is becoming increasingly common due to persistent infections, which can have severe morbidity and mortality consequences. This resistance limits therapeutic options, leading to a clinical situation where existing antibiotics are ineffective in treating certain human infections. The possibility of gene recombination between different bacterial populations is significant, and bacteria seem to acquire genetic resources relatively quickly to thrive in environments that would have otherwise hindered their growth. In order to advance precision healthcare, it is imperative to gain a deeper understanding of the intricate interplay between microbial persisters and the host, which will enable the development of innovative strategies to address this complex issue. Recent research has shown that several underlying mechanisms contribute to this phenomenon. To effectively eliminate persistent bacteria, it is essential to understand how growth-arrested persisters regrow. Gaining knowledge about the internal and external factors, as well as the cellular heterogeneity of persisters and their regrowth, can lead to the development of new therapeutic agents. One potential strategy is to prevent the evolution of tolerance, which could delay the emergence of antibiotic resistance. The emergence of antimicrobial resistance is a major concern, and it is imperative to revive the development of new and effective drugs to combat this deadly condition.
